# Potential Barriers of Patient Involvement in Health Technology Assessment in Central and Eastern European Countries

**DOI:** 10.3389/fpubh.2022.922708

**Published:** 2022-07-28

**Authors:** Maria Dimitrova, Ivett Jakab, Zornitsa Mitkova, Maria Kamusheva, Konstantin Tachkov, Bertalan Nemeth, Antal Zemplenyi, Dalia Dawoud, Diana M. J. Delnoij, François Houýez, Zoltan Kalo

**Affiliations:** ^1^Faculty of Pharmacy, Medical University of Sofia, Sofia, Bulgaria; ^2^Syreon Research Institute, Budapest, Hungary; ^3^Center for Health Technology Assessment and Pharmacoeconomics Research, Faculty of Pharmacy, University of Pécs, Pécs, Hungary; ^4^National Institute for Health and Care Excellence, London, United Kingdom; ^5^Faculty of Pharmacy, Cairo University, Cairo, Egypt; ^6^Erasmus School of Health Policy and Management, Erasmus University, Rotterdam, Netherlands; ^7^National Health Care Institute (Zorginstituut Nederland), Diemen, Netherlands; ^8^EURORDIS: Eurordis, European Organization for Rare Diseases, Paris, France; ^9^Center for Health Technology Assessment, Semmelweis University, Budapest, Hungary

**Keywords:** patient engagement, health technology assessment (HTA), barrier, central and eastern EU countries, potential

## Abstract

Patients' perspectives are important to identify preferences, estimate values and appreciate unmet medical needs in the process of research and development and subsequent assessment of new health technologies. Patient and public involvement in health technology assessment (HTA) is essential in understanding and assessing wider implications of coverage and reimbursement decisions for patients, their relatives, caregivers, and the general population. There are two approaches to incorporating the patients' voice in HTA, preferably used in a mix. In the first one, patients, caregivers and/or their representatives directly participate at discussions in different stages of the HTA process, often at the same table with other stakeholders. Secondly, patient involvement activities can be supported by evidence on patient value and experience collected directly from patients, caregivers and/or their representatives often by patient groups Patient involvement practices, however, are limited in Central and Eastern European (CEE) countries without clear methodology or regulatory mechanisms to guide patient involvement in the HTA process. This poses the question of transferability of practices used in other countries, and might call for the development of new CEE-specific guidelines and methods. In this study we aim to map potential barriers of patient involvement in HTA in countries of the CEE region.

## Introduction

Patients' perspectives are important to identify preferences, estimate values and appreciate unmet medical needs in the process of research and development and subsequent assessment of new health technologies (1). Health technology assessment (HTA) is a multidisciplinary process that uses explicit methods to determine the value of a health technology from different dimensions. Such health technology can be a medical test, device, medicine, vaccine, medical procedure, program, or even a health policy intervention (2). Patient and public involvement in HTA is essential in understanding and assessing wider implications of coverage and reimbursement decisions for patients, their relatives, caregivers, and the general population (1).

Patient involvement is intended to inform all the elements of an HTA from shaping research questions, early dialogues, informing cost-effectiveness models and/or the deliberation process (3, 4). There are two approaches to incorporating the patients' voice in HTA, preferably used in a mix. In the first one, patients, caregivers and/or their representatives directly participate at discussions in different stages of the HTA process, often at the same table with other stakeholders (5). Within this approach, several different methods can be used reflecting different aspects and levels of involvement [e.g., call for written comments, organizing a patient panel, inviting patient(s) to an Advisory Board or focus groups]. Secondly, patient involvement activities can be supported by evidence on patient value and experience collected directly from patients, caregivers and/or their representatives often by patient groups (6). Subsequently, the need for scientific justification of the point of view of patients and society is enhanced by the increasing number of studies to measure not only patients‘ preferences (i.e., time trade-off, standard gamble, etc.) but also patients‘ involvement in the collection of patient‘s reported outcomes (7, 8). Evidence generated through patient preference studies is becoming recognized by HTA organizations as a valuable addition to health technology submissions (9).

The general guide for patient involvement and mix of methods to use should be developed together with patients and fitting the local regulatory environment (2). The method and level of HTA is determined at the national and regional level, along with the method and level of patient involvement. There are European level initiatives such as the European network for Health Technology Assessment (EUnetHTA) jointly assessing selected new technologies and just recently there was a new harmonized regulation on HTA adopted in the European Union (EU), but no EU regulation of HTA applies until January 2025 (10). Thereby, to this date, the approach to patient involvement differs greatly by countries and regions (2, 11, 12). A survey by the European Patients' Forum in 2012 concluded that there are regional differences in the proportion of HTA agencies with and without patient involvement within Europe (13). These findings are in line with results of a survey performed by Health Technology Assessment International's Patient and Citizen Involvement Group (HTAi PCIG) in 2016 (14). The following countries reported patient involvement activities in one or more of their HTA organizations: Australia, Canada, Columbia, England, France, Germany, Italy, Netherlands, Sweden, Taiwan, Poland, Scotland and Wales. Remarkably, there was only one HTA organization from the Central Eastern European (CEE) region reporting on patient involvement activities—the Agency for Health Technology Assessment in Poland. Other CEE countries' HTA organizations did not respond to the survey, thereby there might have been some unreported patient involvement activities in CEE countries. However, the regional difference in response in itself warrants further investigation of patient involvement in HTA in CEE countries.

Compared other regions of Europe, CEE countries are in general at less advanced stages of implementing HTA in spite of the great need for evidence-based resource allocation decisions (15). Some exceptions exist, and a general positive trend can be observed, but the gap between the CEE region and the rest of Europe is still detectable. There are additional—albeit relating—cultural, historical, economic, organizational differences to be taken into consideration when applying good practices of HTA to the CEE context. This is particularly the case for patient involvement in HTA. Patient involvement practices are limited in CEE countries without clear methodology or regulatory mechanisms to guide patient involvement in the HTA process. This poses the question of transferability of practices used in other countries, and might call for the development of new CEE-specific guidelines and methods. However, this requires more insight into patient involvement HTA practices in CEE and the factors that promote or inhibit this. Therefore, in this study we aim to map potential barriers of patient involvement in HTA in countries of the CEE region.

## Methodology

This research was conducted as part of the HTx project. HTx is a Horizon 2020 project supported by the European Union lasting for 5 years from January 2019 (www.htx-h2020.eu). The main aim of HTx is to create a framework for the Next Generation Health Technology Assessment to support patient-centered, societally oriented, real-time decision-making on access to and reimbursement for health technologies throughout Europe. Through Work Package 5, HTx aims to assess transferability aspects of novel HTA methodology from Western Europe (WE) countries to CEE countries and form recommendations. Patient-centered and socially-oriented HTA being in the focus of HTx, patient involvement in HTA was selected as a good practice to be included in such an assessment.

The study was conducted in three phases: (1) a scoping literature review to identify potential barriers of patient involvement in HTA, (2) a workshop with relevant stakeholders from CEE countries and experts from the HTx consortium to identify additional barriers, (3) an iterative process ran throughout these phases by CEE researchers from the HTx consortium deduplicating, merging and categorizing identified barriers.

### Literature Review

The scoping literature review aimed to identify publications discussing potential barriers of patient involvement in HTA. The literature search was conducted through the PubMed database on the 30th of September in 2020, using the combination of the following keywords: patient; public; health technology assessment; HTA; involve; engage. The search was limited to English-language papers published in the past 10 years. Websites of relevant European Commission funded policy research projects (Innovative Medicines Initiative, Horizon 2020) were screened for project deliverables concerning patient involvement in HTA. Additionally we included experts of the field (patient involvement in HTA) that could propose additional peer-reviewed articles deem important but missed by the literature search.

Identified articles were deduplicated and screened first in the title and abstract screening phase, then those included were reviewed in full-text. The following exclusion criteria were used: (1) No abstract; (2) Not English language, (3) Not published in the past 10 years; (4) Not discussing HTA; (5) Not focusing on patient involvement; (6) Not mentioning any barriers of patient involvement in HTA. Identified barriers were extracted and served as a basis for the workshop and the iterative process.

### Webinar

The second step of the study was a live webinar organized for HTx consortium members and CEE stakeholders, including payers, academics, healthcare professionals, industry and patient representatives. The aim of the webinar was to present study results and further identify barriers of patient involvement in HTA from the different perspectives, relevant for the CEE region. Because of the challenges related with the existing COVID-19 pandemic the webinar was held online 0.73 attendees from 12 CEE countries managed to joined the webinar on the 4th of December 2020. All invited attendees got pre-meeting materials for preparations and the interim results of the scoping literature review were presented as a basis for discussion. Based on their expertise and perspective the attendees provided written comments on the most important barriers identified through the literature search process.

### Iterative Process

From September 2020 to January 2021, parallel to the other research phases, the main research team conducted the iterative process. The main research team consisted of eight researchers from the two CEE partners within the HTx consortium coming from different areas of health economics and patient-centered research. Final consensus for the identified barriers was reached after series of regular research team meetings for deduplication, merge, categorization and clarification of the identified barriers.

## Results

**Figure 1:**
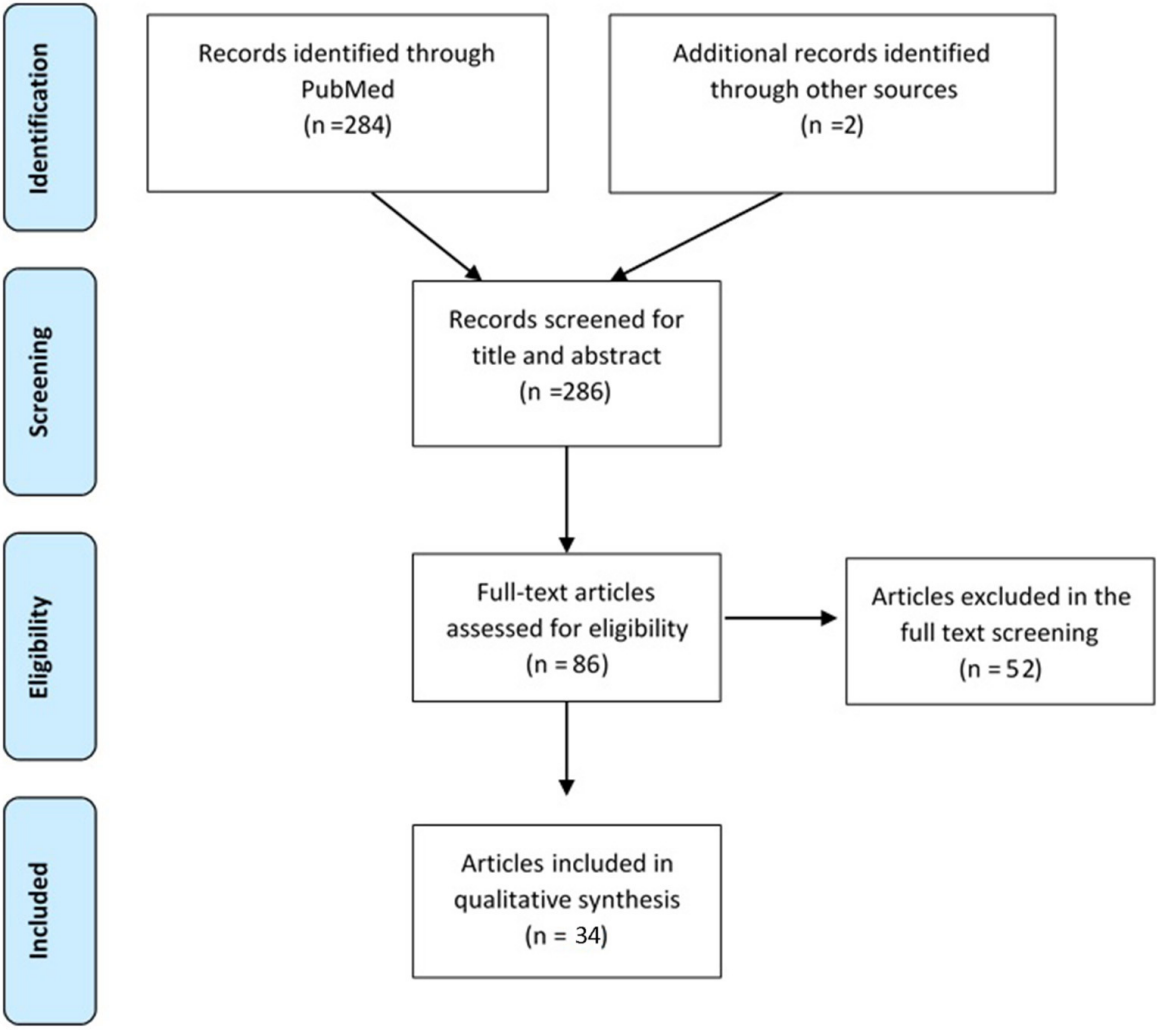
Flow-diagram of articles screened in the scoping literature review.

Thirty-two (*n* = 32) published scientific articles and two gray literature sources meeting the predefined criteria were identified by the scoping literature review (Figure 1).

The list with the included papers (*n* = 34) with basic characteristics can be found in Table 1.

**Table 1:** List of papers included in the scoping literature review.

**Publication**	**Objective**
Facey et al. (16)	To outline key concepts for complementary ways in which HTAs could be strengthened by taking account of patients' perspectives.
Fonsdal et al. (17)	To analyse systematic reviews which considered implementation and monitoring strategies to optimize technology uptake and use, and the implications of such strategies for HTA used in decision-making processe and ways how to engage patients in the process.
Gangon et al. (18)	To review the international experience of patient and public involvement in the field of HTA.
Menon and Stafinski (19)	To analyse findings from peer-reviewed and “gray” literature, and discussions with key informants to determine potential roles for patients and the public in HTA and coverage decision-making and existing roles for both groups in jurisdictions.
Danner et al. (20)	To introduce the analytic hierarchy process (AHP) as a patient preference elicitation method in HTA.
Gangon et al. (21)	To (1) validate a reference framework for exploring the relevance and applicability of various models of patient involvement in HTA, (2) implement strategies that involve patients (including close relatives and representatives) at different stages of the HTA process, (3) evaluate intervention processes, and (4) explore the impact of these interventions on (a) the applicability and acceptability of recommendations arising from the assessment, (b) patient satisfaction, and (c) the sustainability of this approach in HTA.
Cavazza and Jommi (22)	To investigate stakeholder involvement by HTA Organizations in France, Spain, England and Wales, Germany, Sweden, and the Netherlands and to examine factors this involvement depends on.
Drummond et al. (23)	To explore approaches in terms of both policy and methods in engaging patients and public and other different perspectives in assessing the added value of health technologies.
Haley et al. (14)	To obtain further information from members of the International Network of Agencies for Health Technology Assessment (INAHTA) on the involvement of consumers in their programs.
Gagnon et al. (24)	To (1) set up interventions to promote patient participation in three stages of the HTA process: identification of HTA topics, prioritization, and development of the assessment plan of the topic prioritized; and (2) assess the impact of patient participation on the relevance of the topics suggested, the prioritization process, and the assessment plan from the point of view of patients and other groups involved in HTA.
Mühlbacher, (25)	The paper stipulates that decision criteria must be relevant to the patient. Patients might value different clinical end points differently. The paradigm of patient centeredness aims to ensure that the interests of patients are adequately taken into account. Therefore, patient segmentation is the key to patient-centered healthcare systems. Patient-centric health technology assessment must inform decision makers about patients' preferences.
Dipankui et al. (26)	To evaluate two patient involvement strategies (consultation and direct participation) in the assessment of alternative measures to restraint and seclusion among adults in short-term hospital wards (in psychiatry) and long-term care facilities for the elderly.
Abelson et al. (27)	To describe the development and outputs of a comprehensive framework for involving the public and patients in a government agency's HTA process.
Husereau et al. (28)	Innovation in health technology assessment (HTA) is needed to support changing health system environments and to help provide access to valuable innovation under fiscal constraint. The objective of the paper is to identify through scoping and explored through deliberation at a meeting of industry and HTA leaders.
Hämeen-Anttila et al. (29)	To discover ways to involve patients in HTA and clinical practice guidelines processes, to describe challenges, and to find ways of informing patients about HTAs and CPGs in Finland.
Iskrov and Stefanov (30)	To analyse what needs to be done in the proves of public health reimbursement decision-making through the perspective of patients and other stakeholders.
Weeks et al. (31)	To advance understanding of the range of evaluation strategies adopted by HTA organizations and their potential usefulness through the perspective of Health Technology Assessment International's (HTAi's) Patient and Citizen Involvement Group (PCIG).
Wortley et al. (32)	To describe community views and perspectives on public engagement processes in Australian HTA decision making.
Iskrov et al. (33)	Authors aim to provide solutions for optimisation of assessment and appraisal of new rare disease therapies is a fundamental issue in rare disease health policy through establishment of consensus-building tools to foster cooperation and collaboration through consensus-building tools (e.g., focus group discussion).
Addario et al. (34)	To explore the varying definitions of patient value and make positive recommendations for working together to strengthen the patient voice in this area.
Scott and Wale (35)	To analyse through a survey the views of patient advocates who were members of patient organizations known to be engaged in the process of HTA or evidence-based practice.
Wale et al. (36)	To provide arguments why patient involvement should be prioritized by those HTA agencies that do not yet involve patients: (1) from a patients' rights perspective, (2) based on patient and community values, (3) centering on evidentiary contributions, and (4) from a methodological perspective.
Facey et al. (37)	To analyse how the needs, preferences and experiences of the patients could be used to support decision making.
Simpson et al. (38)	With this paper authors aim to report on the experiences, benefits, and challenges of patient and public involvement and engagement (PPIE) from a publicly funded early awareness and alert (EAA) system in the United Kingdom through identification, filtration, prioritization, early assessment, and dissemination.
Boudes et al. (39)	To analyze through qualitative survey the stakeholder expectations on patient engagement in medicines development and during the life cycle of a product.
Hunter et al. (1)	To analyse the formal publication of the HTA guidance text with discussion about recent progress in, and continuing barriers to, patient involvement in HTA.
Janssens et al. (40)	To identify barriers for transitioning patient involvement from theory to practice in the lifecycle of medicines.
Paradigm, (41)	To provide perspectives from HTA organizations on the potential to develop patient involvement in Early Dialogue/Scientific Advice processes.
Littlejohns et al. (42)	To analyse the framework of multidisciplinary collaboration in England and in New Zealand using a mixed-methods approach in terms of finding possible solutions on how to engage the competence of the representatives from the different organizations included in the process.
Wilking et al. (43)	To analyse how early and regular dialogue between all stakeholders including regulators, payers, patient stakeholders and industry is required to improve the situation could facilitate decision making in access to oncology therapies.
Babac et al. (44)	To examine whether patient perspectives are considered as part of early benefit assessments for rare diseases and how patient perspectives are methodologically elicited and presented.
Single et al. (3)	To promote further discussion about the ways in which patient involvement can impact HTAs, studying particular cases in-depth, using stories told by people who lead the practice in HTA bodies.
Wale and Sullivan (45)	To explore how written and oral patient involvement in two HTAs was reported on in publicly available final recommendations and discussion summaries of appraisal committees from three HTA bodies. The authors aimed to gain insights into how patient input was utilized by appraisal committees to better understand the goals of patient involvement and how these are being achieved.
Paradigm, (46)	To have a common framework that enables structured, effective, meaningful, ethical, innovative, and sustainable patient engagement and demonstrates the “return on engagement” for all players.

Included articles focused on (1) incentives and expectations of patient involvement in HTA (2) different methods of inclusion (3) methods of impact assessment (4) mapping current practices and actual impact. All included articles mentioned at least one potential barrier of patient involvement in HTA. The scoping review shows that, after 2015, there is a significant rise in the published articles looking into challenges and possible barriers of patient involvement in HTA.

The final set of potential barriers came along as a result of the scoping review, workshop and the parallel iterative process. Twenty-five (*n* = 25) potential barriers were identified and investigated. First, barriers were classified based on whose perspective they relate to from the two main stakeholder groups included in these patient involvement activities in HTA—payers/HTA organization representatives and patients. Fourteen (*n* = 14) barriers appeared from the side of payers/HTA bodies and eleven (*n* = 11) from the side of patients (Table 2). Then to ease understanding these barriers were grouped as follows: (1) payer/HTA side: Limited willingness to involve patients; Conflict of interest and confidentiality; Difficulties to finding the “right” patient; Lack of human resources at relevant public institutes; Not knowing how to involve patients. (2) Patient side: Lack of understanding the decision context; Lack of knowledge and guidance of evidence-based advocacy; Lack of resources to be spent on meaningful patient representation; Lack of ethical guidance for representativeness.

**Table 2:** Potential barriers of patient involvement in HTA in CEE countries.

	**Categories**	**Potential barriers**
**PAYER/HTA BODY PERSPECTIVE**	Limited willingness to involve patients	- Limited impact of societal factors on pricing and reimbursement decisions (i.e., the reimbursement decision is evaluated only from the payer perspective per legal framework) - Lack of understanding of the added value of involving patients in the HTA process - General lack of trust in the objectivity and relevance of “patient stories” (e.g., fear of emotional aspects negatively affecting the decision-making process) - Patient involvement in HTA is not mandatory/is not mentioned in the local HTA guideline
	Conflict of interest & confidentiality	- Fear of potential conflict of interest issues due to industry funding of patient organizations - Fear of the violation of confidentiality by patient representatives
	Difficulties to finding the “right” patient representative	- Lack of support and supporting tools (e.g., registries or network) to help patient recruitment - Difficulty to identify representatives from the disease area needed (e.g., some patient communities may have “louder voices” than others) - Lack of understanding of different patient roles (whether the patient is representing their own views or their patient community's) - Patient representatives might not be representative of the whole patient community in terms of socioeconomic status and other basic characteristics (e.g., higher educated, somewhat younger, health-literate patients tend to take on these roles)
	Lack of human resources at relevant public institutes	- Fear of the patient involvement process needing too much support time amidst the tight HTA decision timelines - Payer or HTA organizations do not have enough human resources/time to involve patients (even though they would intend to)
	Not knowing how to involve patients	- Lack of experience/training/skills from the HTA and payer organizations' side in knowing how and when to incorporate patient perspectives - Lack of local (regional or country-specific) guidelines on best practices of patient involvement to HTA
**PATIENT PERSPECTIVE**	Lack of understanding the decision context	- Patient representatives' lack of basic knowledge in HTA - Patient representatives' lack of knowledge of the local regulatory processes including how they can get involved - Patient representatives' lack of knowledge in the medical language - Patient representatives do not speak/understand English which limits the amount of information (training, other countries' experience, scientific literature) they can access
	Lack of knowledge and guidance of evidence-based advocacy	- No methodological guidance to support the activities of patient organizations in collecting data (e.g., survey) valuable for HTA - Patients' lack of experience in searching and/or interpreting information from independent resources (i.e., scientific articles)
	Lack of resources to be spent on meaningful patient representation	- No fair compensation for time offered and logistics issues (e.g., traveling time and costs, documents not sent on time for review, preparatory calls or meetings during working hours) -General lack of capacities due to financial constrains
	Lack of ethical guidance for representativeness	-No clear rules on how to represent a patient community and how to distinguish it from representing their individual patient perspective plus confidentiality prevents patient representatives from discussing/sharing views with others before attending HTA procedures/meetings

Most of the barriers coming from the perspective of the payers/HTA bodies are related to the lack of defined rules how and when to include patients‘ representatives which increases the risk for lack of trust and fear to include patients in the HTA process.

From patients‘ perspective the most of the identified barriers were associated with lack of sufficiently explained methodology for the patient‘s role in the HTA process—lack of HTA and regulatory processes knowledge, medical language knowledge, etc. This could be attributed to the lack of organized programs in the health care systems at all for patient engagement in collecting patient reported outcomes and real world evidence.

## Discussion

Patient engagement in HTA is considered as a transformative strategy which still needs to be adopted in most European countries (47). To a certain extent, the difficulties identified here are not specific for patient engagement in HTA nor for CEE countries, but emerge in patient involvement in healthcare decision-making in general and worldwide. For example, De Graaff et al. elaborate on questions such as whom to involve, how to involve patients and the public, and how to value their input in healthcare decision-making. They argue that more attention should be paid to the work needed for patient and public involvement to explain the gap between expected and current practice (48). Specific barriers such as time investment and budgetary constraints have been reported by Wiering et al. in their study of patient involvement in the development of Patient-Reported Outcome Measures (8). Peeters et al. describe patient organizations' involvement in quality improvement projects in The Netherlands as vulnerable, because of insecure funding and lack of negotiating power (49). Wiig et al. mapped patient involvement in regulatory practice in Norway, England, the Netherlands, and Australia. Their study pointed to several difficulties, such as how to incorporate patients' input, lack of willingness of patients to be involved, time and costs required, barriers related to organizational procedures, and dealing with emotions (50). Finally, in their analysis of decision-making in Dutch HTA practice, Moes et al. point to the risk of so-called epistemic injustice that occurs if patients are being “frustrated in their capacity to be heard and make themselves understood” (51).

Some studies also raise the questions of how we can build trust, partnership and collaborative working environment and what tools and knowledge could be effective to bring together patients/public and policy makers (52, 53).

However, despite the fact that several difficulties are of a more general nature, there are specific barriers for implementation in CEE countries, that call for specific guidelines of patient engagement (37). These specific barriers are, for instance, uncertainty in the role of civil societies, including patient organizations in political processes, or more pressing budgetary constraints. The development of CEE specific guidelines can build on general guidelines on patient involvement in HTA incorporating learnings from the identified barriers. Regarding patient engagement and patient preferences in HTA, many approaches were identified and listed as good practice documents, tracing possible mechanisms for inclusion of the patients in the decision-making process. These approaches also aim to establish guidance on different stakeholder involvement, identify emerging strategies and state of art methods do overcome challenges related to the patient engagement in the HTA process (25, 54–68). Notably, the European Patients' Academy on Therapeutic Innovation (EUPATI) published four guidance papers for patient involvement, one of which was about HTA. This guideline includes suggested working practices for both HTA agencies and patient organizations, as well as suggested patient involvement activities for general HTA processes and individual HTAs. Additionally, both EUnetHTA and Heath Technology Assessment International's Patient and Citizen Involvement Interest Group (HTAi PCIG) regularly publishes and updates guides and templates to aid patient involvement practices (69–71).

To our best knowledge, this is the first published study focusing on barriers of patient involvement in HTA specifically in CEE countries. However, a report with similar scope was identified through the gray literature review. The PARADIGM project was a public-private Innovative Medicines Initiative (IMI) partnership active between 2018 and 2020 (72). The project's mission was to provide a unique framework that enables structured, effective, meaningful, ethical, innovative, and sustainable patient engagement and demonstrates the “return on the engagement” for all players. Within the sustainability assessment of the patient engagement roadmap developed through the project, a workshop was held for stakeholders across the CEE region (37). Four end goals were identified for the European patient engagement landscape: (1) Establish an ethical, trust-based collaboration among all patient engagement stakeholders involved in medicines development; (2) Secure inclusive and diverse patient engagement; (3) Embed patient engagement in the mind-set and at every step and across organizations; (4) Ensure dedicated leadership and operational time, resources and funding. Forty-three (*n* = 43) barriers were identified challenging the reach to these four end goals. The concept was tested through a CEE workshop, however, the deliverable did not focus on CEE-specific barriers and the scope was research and development, not HTA.

A recent systematic literature review by Gagnon and colleagues (5) in 2021 aimed to summarize current evidence on patient and public involvement in HTA and to propose a framework to assess its impact. Thirty-one (*n* = 31) studies were included described in 36 publications. One study reporting on the a HTA case study of palliative care involved two CEE countries amongst others, Lithuania and Poland (73). All other studies had settings other than the CEE region; most of the included studies were conducted in Canada, followed by Italy, England, Germany and Finland, Austria, Ireland, Scotland, South Korea and Spain. Barriers published in the paper are in line with our findings and there are certain limitations of our research. Firstly, in terms of methodology, a non-systematic approach was taken when reviewing the literature and we limited our search to articles published in English. There is a chance some papers are missed from our review Secondly, CEE stakeholders included in the webinar had the chance to comment on the list of barriers and propose missing ones, however, the collated barriers were not ranked in terms of priority. We plan to continue our research with this step. Thirdly, our study did not cover potential action plans and recommendations to address the identified barriers. We deem this step to be crucial in advocating for a change of patient involvement practices, thereby our future steps will cover this aspect as well.

Next steps of the research include the ranking of identified barriers and proposing solutions by a broad CEE stakeholder group. The results of the latter studies are to be published in the future. All future studies are planned to be published to serve as a tool for meaningful patient involvement in HTA and pricing and reimbursement decisions in the CEE region.

## Conclusion

In conclusion, there is a lack of published evidence on real-world extent of and barriers to patient involvement in HTA in CEE countries. On the other hand, there are available guidelines and best practices that could be adopted in these settings. Twenty-five potential barriers of patient involvement in HTA were identified relevant for the CEE context. These will need further investigation to assess their relative importance and develop potential solutions and recommendations for action to address them.

## Data Availability Statement

The raw data supporting the conclusions of this article is available upon request from authors.

## Author Contributions

MD performed the scoping review. MD and IJ drafted the manuscript. MK, ZM, KT, BN, AZ, DDa DDe, FH, and ZK reviewed the paper. All authors have provided valuable contributions to the manuscript, read, and approved the final manuscript.

## Funding

The HTx project has received funding from the European Unions Horizon 2020 research and innovation program under grant agreement N° 825162. This dissemination reflects only the author's view and the Commission is not responsible for any use that may be made of the information it contains.

## Conflict of Interest

Authors IJ, AZ, BN, and ZK are employed by Syreon Research Institute. At the time of the study IJ was the President of the European Patients' Forum Youth Group and a Board of Trustees member at the EUPATI Foundation. The remaining authors declare that the research was conducted in the absence of any commercial or financial relationships that could be construed as a potential conflict of interest.

## Publisher's Note

All claims expressed in this article are solely those of the authors and do not necessarily represent those of their affiliated organizations, or those of the publisher, the editors and the reviewers. Any product that may be evaluated in this article, or claim that may be made by its manufacturer, is not guaranteed or endorsed by the publisher.
